# Methionine ameliorates intestinal injury in senescence-accelerated mouse prone-8 mice by reducing sulfate-reducing bacteria and enhancing barrier function

**DOI:** 10.3389/fnut.2025.1698518

**Published:** 2025-10-20

**Authors:** Ying Wu, Yong Zhang, Min Zhou, Peng Liu, Xin Rao, Yong Zhang, Mantian Mi

**Affiliations:** ^1^Research Center for Nutrition and Food Safety, Chongqing Key Laboratory of Nutrition and Health, Institute of Military Preventive Medicine Third Military Medical University (Army Medical University), Chongqing, China; ^2^Department of Nutrition, The Sixth Medical Center, Chinese PLA General Hospital, Beijing, China; ^3^PLA Air Force Medical Center, Beijing, China; ^4^The Fourth Medical Center of PLA General Hospital, Beijing, China

**Keywords:** methionine, intestinal injury, SAMP8 mice, sulfate-reducing bacteria, barrier function

## Abstract

**Objective:**

Age-related intestinal barrier dysfunction is a key factor leading to systemic inflammation. Previous studies have found that methionine and its metabolites play a role in anti-aging, but the specific effects on the intestines of aging mice remain unclear. This study aims to explore the effects of different doses of methionine in the diet on intestinal integrity and gut microbiota, and to clarify its potential mechanism in a mouse model of accelerated aging (SAMP8).

**Method:**

SAMP8 mice were selected and divided into three groups, each receiving a Methionine-restricted diet (0.17%Met), normal (0.86%Met), or Methionine-supplemented diet (1.64%Met) for 4 weeks. And SAMP1 mice were used as the control. The intestinal barrier function was evaluated by detecting the levels of LPS, IFABP and Zonulin in serum through ELISA. The integrity of colon tissue, the expression of tight junction proteins (ZO-1 and Occludin) and inflammatory signaling pathways (TLR4/NF-κB) were evaluated by histology, immunofluorescence and Western blot. The composition of the gut microbiota was analyzed by 16S rRNA sequencing, and the levels of hydrogen sulfide (H₂S), sulfomucin in the intestine and the expression of genes related to mucus sulfation were quantitatively detected.

**Result:**

Methionine-supplemented diet (1.64%Met) significantly improved intestinal aging. Specifically, it is manifested as reducing the expression of cellular senescence markers p16 and p21, lowering the levels of LPS, IFABP and zonulin in serum, restoring the disordered colon structure, and upregulating the expression of tight junction proteins (ZO-1, Occludin). The pro-inflammatory effect of a methionine-supplemented diet on the TLR4/NF-κB pathway reduces the production of H₂S in the intestine. In addition, Methionine-supplemented diet reshaped the gut microbiota, increasing the abundance of beneficial bacterial genera (such as *Parabacteroides*) while reducing the abundance of H₂S-producing bacteria (such as *norank_f__Desulfovibrionaceae*). This change in the microbial community is closely related to the concentration of methionine intake and also associated with the recovery of intestinal sulfation, manifested as an increase in the expression of sulfattransferases (such as *Papss2*) and an increase in the production of sulfomucin. On the contrary, a methionine-restricted diet increased the abundance of *norank_f__Desulfovibrionaceae*, exacerbating gut microbiota imbalance and barrier dysfunction.

**Conclusion:**

A methionine-supplemented diet within the safe range significantly alleviates aging-induced intestinal barrier dysfunction by regulating the gut microbiota, inhibiting H₂S-producing bacteria, and restoring the host’s intestinal sulfation capacity. A new microbiota- sulfation axis pathway was revealed, which promotes the metabolism of toxic sulfur substances related to the microbiota (such as H_2_S, indoxyl sulfate, etc.), and methionine supplementation was proposed as a promising nutritional strategy to promote intestinal health and alleviate aging-related pathological changes.

## Introduction

Aging is a complex biological process characterized by the gradual decline in the function of tissues and organs, accompanied by an increased risk of various age-related diseases. The global population aged 65 and above is 761 million (10%), and this number is expected to reach 1.6 billion by 2050 (1). With the aging of the Chinese population, the incidence of age-related diseases such as Parkinson’s disease (PD), Alzheimer’s disease (AD), and malignant tumors continues to rise, imposing a heavy economic burden on the nation and patients’ families (2, 3). Although many drugs such as metformin, resveratrol, and rapamycin have been proven to have anti-aging effects, they have not been widely promoted due to high costs, difficulty in extraction, and severe side effects (4–8). Therefore, in response to the reality of population aging, finding or developing dietary nutrients with anti-aging properties and exploring their mechanisms of action have become current research hotspots.

The intestine, as the largest interface between the body and the external environment, plays a crucial role in maintaining internal homeostasis. However, the gradual degradation of the intestinal barrier during aging has become a significant trigger for various age-related diseases, including inflammatory bowel disease, chronic inflammation, and metabolic disorders (9–11). Recent studies have shown that age-related intestinal barrier dysfunction is mainly manifested by increased permeability, disordered intercellular junction structures, and reduced repair capacity (12). Additionally, the integrity of the intestinal barrier is a highly accurate predictor of mortality in model organisms (13).

Dietary nutrients are not only the material basis of life but also serve as “chemical probes” and “effector molecules” that regulate cellular signaling and physiological functions. Research indicates that dietary amino acids can not only compensate for the loss of amino acids during aging but also regulate intestinal homeostasis, playing a crucial role in maintaining gut health and preventing intestinal diseases (14). Methionine, as an essential amino acid, plays a central role in protein synthesis, methylation metabolism, and antioxidant defense (15, 16). Studies have found that methionine and its metabolites (such as S-adenosylmethionine, SAM) can enhance the proliferation and differentiation capabilities of intestinal stem cells (ISC) by activating the Wnt/*β*-catenin pathway, thereby repairing damaged intestinal epithelium (17, 18). Additionally, glutathione (GSH) generated from methionine metabolism is an important endogenous antioxidant that can alleviate oxidative stress damage associated with aging (19–21). Some studies have reported that Met supplementation can increase the flux through the transsulfuration pathway (TSP), thereby improving the cognitive function of subacute aging mice (22). Supplementing cysteine (such as *N*-acetylcysteine) can restore glutathione levels, reduce oxidative damage, and improve muscle function, inflammation, and appetite in aged mice and rats (23). Selenomethionine (SeMet) has been shown to alleviate ischemia–reperfusion-induced intestinal injury by inhibiting the Bax/Caspase apoptosis pathway (24). Furthermore, in methionine-restricted Apcmin+/− mouse colon tissues, mucin Muc2 is reduced and the transport capacity of colonic sulfomucin is decreased. Conversely, enhancing methionine cycle flux can alleviate intestinal cell senescence and tissue aging through metabolic-epigenetic regulation, thereby affecting intestinal barrier function (25, 26). Although evidence has emerged indicating the potential of methionine in protecting the intestinal barrier, its specific mechanisms of action in aging-related intestinal damage remain to be systematically elucidated. This study aims to go beyond the direct effects on the host and, for the first time, systematically explore how methionine, as a nutritional regulatory molecule, indirectly regulates the host barrier by reshaping the sulfur metabolism of the gut microbiota, thereby revealing a novel “diet-microbiota-host” interaction pathway. Based on this, we hypothesize that increasing dietary methionine levels within a safe range can effectively reverse aging-induced intestinal barrier dysfunction by inhibiting sulfate-reducing bacteria and enhancing the host’s mucin sulfation capacity. Using an accelerated aging mouse model (SAMP8), we designed a dietary intervention experiment with different doses of methionine. A variety of techniques, including serum biomarker detection, histopathological analysis, immunofluorescence, Western blotting, real-time quantitative PCR, 16S rRNA sequencing, and assessment of the spatial distribution of fecal microbial communities, were employed to thoroughly investigate the effects of methionine on intestinal structural integrity, tight junction protein expression, inflammatory pathway activation, gut microbiota composition, and metabolic products (such as H₂S). This approach aims to clarify the potential mechanisms underlying its improvement of aging-related intestinal barrier function and to explore safe and effective nutritional intervention strategies to promote intestinal health and delay the aging process.

## Materials and methods

### Animals and grouping

All animal experimental protocols in this study were approved by the Ethics Committee of the Army Medical University (Approval No.: AMUWEC20211120). SAMP8 mice and SAMR1 mice (purchased from Beijing Zhong’an Funeng Biotechnology Co., Ltd.) were used in the experiment. Before the experiment, male mice were acclimatized in a standard environment for 2 weeks. The standard environmental conditions were: 12-h light–dark cycle, temperature 22 ± 3 °C, relative humidity 50% ± 15%, and free access to food and water. Subsequently, SAMP8 mice were randomly divided into four groups: Methionine Restricted Group (MR + SAMP8 group, 0.17%), Methionine Normal Group (MN + SAMP8 group, 0.86%), Methionine Supplemented Group (MS + SAMP8 group, 1.64%), and normal control group (SAMR1 group, 0.86%). Each group of mice was fed the corresponding diet for 4 weeks. At the end of the experiment (day 28), fecal samples were first collected from the mice, quickly frozen, and stored in a −80 °C freezer for later use. Subsequently, mice were anesthetized by continuous inhalation of 2–3% isoflurane, and blood was collected from the retro-orbital venous plexus, followed by immediate euthanasia by cervical dislocation. The collected blood samples were centrifuged at 3000 g for 15 min at 4 °C, and the separated serum was stored in a −80 °C freezer for subsequent analysis. The entire experimental process ensured the consistency of sample collection and the accuracy of analytical results.

### Detection of intestinal injury markers

According to the kit instructions, the concentrations of LPS, IFABP, and Zonulin in serum, as well as the concentration of H_2_S in colon tissue, were measured and subjected to statistical analysis (27, 28).

### Pathological histomorphological detection

Twon cm of proximal colon tissue was taken, fixed with 4% paraformaldehyde, and 4 μm sections were prepared after conventional paraffin embedding (29). Hematoxylin–eosin (HE) staining, ferric diamine-alxin blue (HID-AB) staining and immunohistochemical staining were performed, respectively (30). The stained sections were observed using a Leica DM750 optical microscope (Leica Microsystems, Wetzlar, Germany), and images were captured with a 20 × objective lens. Image recording is carried out using the Leica MC170 high-definition digital imaging system, and fluorescence microscope images are synchronously collected (31). The pathological scoring of the colonic tissues was performed by a pathologist in a single-blind manner based on the HE results, with the scoring criteria referenced from previous studies: 0 points: no obvious inflammation; 1 point: mild inflammation with scattered mononuclear cell infiltration; 2 points: moderate inflammation with multifocal infiltration; 3 points: severe inflammation with vascular congestion and marked thickening of the vascular wall; 4 points: leukocyte infiltration inflammation (32).

### FISH (fluorescence *in situ* hybridization) detection of intestinal bacteria

After the tissue samples were cut, they were immediately rinsed rapidly with PBS, then soaked in Carnoy fixative (Servicebio) for more than 12 h and stored at 4 °C. The fixed tissue was dehydrated by gradient alcohol, transparent with xylene and embedded in paraffin. The paraffin blocks were cut into 4 μm thin slices, treated with dewaxing solution, and then rehydrated with 85 and 75% alcohol for 5 min, respectively. Finally, they were soaked in DEPC water. Antigen extraction was carried out using a repair solution containing proteinase K, followed by rinsing with distilled water and washing three times with PBS (each time for 5 min). The sections were hybridized with the pan-bacterial probe EUB338 at 46 °C. The probe hybridization buffer (900 mM NaCl, 20 mM Tris–HCl [pH 7.5], 0.02% SDS, 10% [w/v] dextran sulfate). The concentration of 1% [w/v] blocking normal donkey serum was 8.0 pmol/μl, and the hybridization was overnight. After hybridization, wash with gradient citrate buffer at 40 °C for 10 min and DAPI dark staining for 8 min. Finally, images were captured using a confocal microscope (Nikon E-C2 from Japan).

### Real-time quantitative PCR

Total RNA was extracted from intestinal tissues using RNAiso Plus reagent (Takara Bio, Kusatsu, Japan). Subsequently, total RNA was reverse transcribed into cDNA using the PrimeScript RT Master Mix kit (Takara Bio, Shiga, Japan), and the operation was carried out strictly in accordance with the manufacturer’s instructions. qRT-PCR was performed on the qTower 2.2 real-time PCR system (Analytik Jena, Thuringia, Germany) using SYBR Premix Ex Taq II (Takara Bio, Japan). Taking GAPDH as the internal reference gene, the relative expression level of mRNA was calculated by the 2^-ΔΔCt method.

### Western blot

The colon samples were homogenized with RIPA lysis buffer and then centrifuged at 4 °C and 10,000 × *g* for 10 min to collect total protein. The protein concentration was determined using the BCA protein Assay Kit (Thermo Fisher Scientific, USA). Subsequently, equal amounts of protein (50 μg per well) were separated by 10% SDS-polyacrylamide gel electrophoresis and electrotransferred to polyvinylidene fluoride (PVDF) membranes (Millipore, Marlborough, MA, USA). Seal the membrane with a 5% skimmed milk solution (containing 1 times TBST) at room temperature for 1 h. Subsequently, the membrane was combined with TLR4 (1:1000, 66,350-1-IG, Proteintech, USA), Phospho-NF-κB (1:1000, AP1294, Abclonal, China), and NF-κB (1:1000, A2547, Abclonal, China), Occludin (1:1000, A24601, Abclonal, China), AHR (1:2000, 67,785-1-IG, Proteintech, USA), OATP2B1(1:1000 55,180-1-AP, Proteintech, USA), p16 (1:1000, 10,883-1-AP, Proteintech, USA) and p21 (1:5000, A22460, Abclonal, China) *β*-actin (1:1000) Incubate with AF0003, Beyotime Biotechnology.

### 16S rRNA sequencing of gut microbiota

After collecting the fresh feces of mice, they were immediately quick-frozen with liquid nitrogen and stored at −80 °C. Total microbial DNA was extracted using the FastPure Stool DNA Isolation Kit (MJYH, Shanghai, China). The DNA mass and concentration were detected by 1.0% agarose gel electrophoresis and NanoDrop 2000 spectrophotometer (Thermo Scientific, USA), respectively, and then stored at −80 °C for future use. The hypervariable region of the V3-V4 gene of bacterial 16S rRNA was treated with primers 338F (5’-actCCTACGGGAGGCAGCAGCAG-3’) and 806R (5’-GGACTACHVGGGTWTCTAAT-3’). Amplification was carried out on the T100 Thermal Cycler PCR instrument (BIO-RAD, USA). The PCR system (20 μL) includes: 5 × Fast Pfu buffer 4 μL, 2.5 mM dNTPs 2 μL, upstream and downstream primers (5 μM) 0.8 μL each, Fast Pfu polymerase 0.4 μL, template DNA 10 ng, ddH₂O replenished to 20 μL. PCR procedure: Pre-denaturation at 95 °C for 3 min; Denaturation at 95 °C for 30 s, annealing at 55 °C for 30 s, and extension at 72 °C for 45 s, totaling 27 cycles. Finally, extend for 10 min at 72 °C and terminate at 4 °C. After the PCR products were separated by 2% agarose gel electrophoresis, the target bands were cut. The PCR Cleanup Kit (YuHua, Shanghai, China) was used for purification, and quantitative analysis was performed using a Qubit 4.0 fluorometer (Thermo Fisher Scientific, USA).

### Analysis of the fecal microbiome in mice

The total genomic DNA of microorganisms in fecal samples was extracted using the PF Mag-Bind Stool DNA Kit (Norcross, GA, USA). For the V3-v4 hypervariable region of the 16S rRNA gene, primers 338F (5’-ACTCCTACGGGAGGCAGCAG-3’) and 806R (5’-GGACTACHVGGGTWTCTAAT-3’) were used. Amplification reactions were carried out on the ABI GeneAmp® 9,700 PCR instrument (Thermo Fisher Scientific, CA, USA). The amplification products were sent to the Illumina MiSeq platform (San Diego, CA, USA) for sequencing analysis.

For the obtained original read segments, quality control work was first carried out using the fastp software, and then with the help of the FLASH software. With 97% sequence similarity as the threshold, the UPARSE v11 software Clustering of sequences to generate operational taxonomic units (OTUs) At the same time, remove the chimeric sequences within it.

Using Mothur v1.30.2 software Introducing mothur: Open-source, platform-independent, community-supported software for describing and comparing microbial communities. Calculate the *α* -diversity index, including the observed ASV, Chao1 index, Shannon index and Good’s coverage. Principal coordinate analysis based on Bray-Curtis distance (PCoA) was accomplished via the Vegan v2.4.3 software package. The identification of differential microbiota was performed using STAMP v2.0.0 software and the LEfSe method (setting LDA score > 2.0 and FDR < 0.05) was used to screen microbial biomarkers.

### Statistical analysis

Data are expressed as mean ± standard deviation. Statistical analysis was performed using GraphPad Prism 8.0.2 (Insightful Science, San Diego, CA, USA). Student’s t-test was used for comparison between the two groups, and one-way analysis of variance (ANOVA) combined with Bonferroni *post hoc* test was used for differences among multiple groups. A *p* value < 0.05 was regarded as statistically significant. Significance is marked as follows: ns indicates no significant difference; *p < 0.05; **p < 0.01; ***p < 0.001; ****p < 0.0001.

## Result

### Methionine-supplemented can improve the intestinal barrier function of SAMP8 mice and alleviate age-related phenotypes

**Figure 1:**
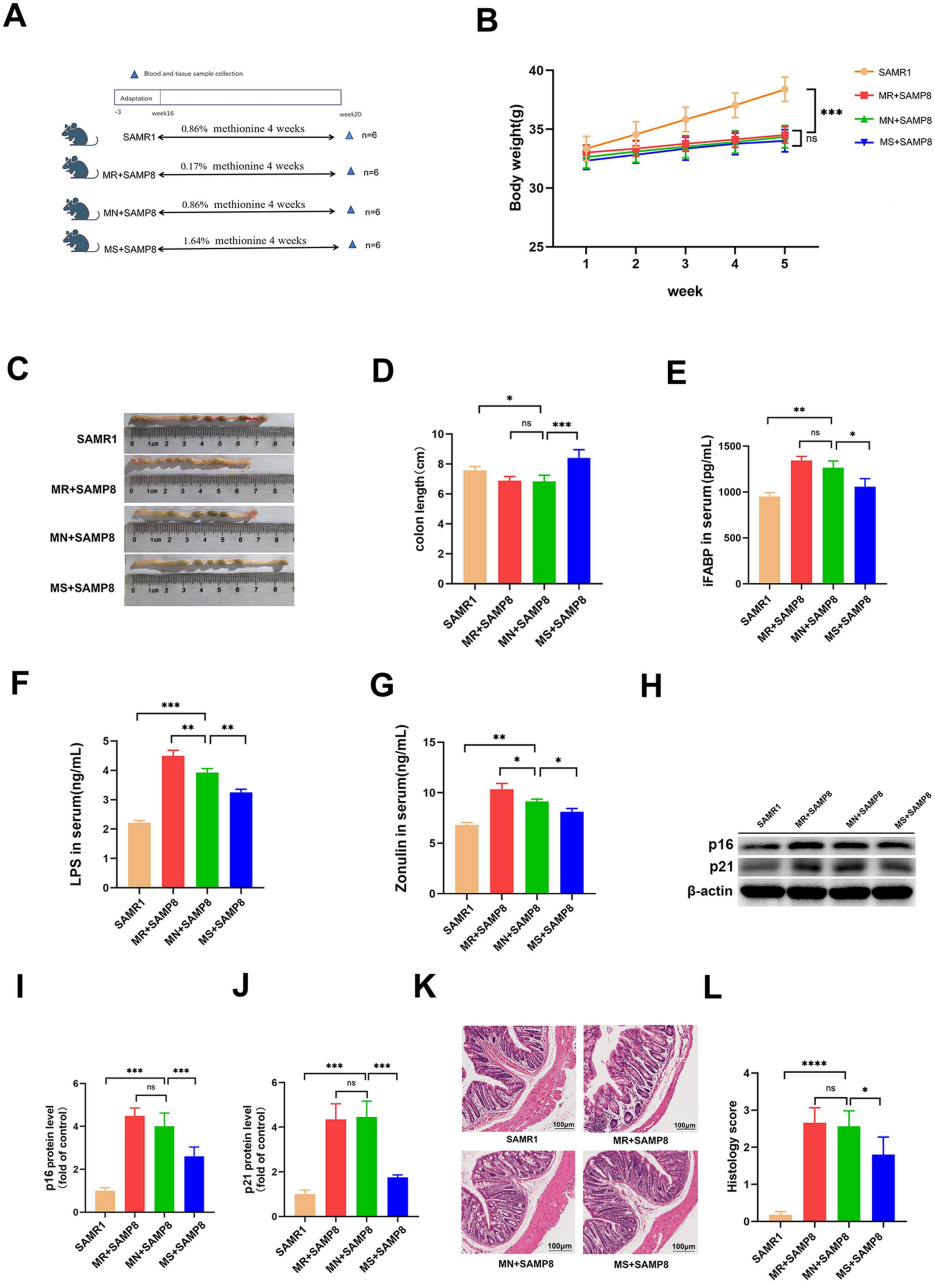
Effects of different dietary methionine levels on body weight, colon length, and intestinal barrier function in mice. **(A)** Dietary methionine dose-escalation protocol (*n* = 6 per group). **(B)** Changes in mouse body weight (*n* = 6 per group). **(C,D)** Changes in mouse colon length (*n* = 6 per group). **(E)** Serum iFABP level **(F)** Serum LPS level. **(G)** Serum Zonulin level **(H–J)** representative Western blots and quantified protein levels of p16, and p21 in colon (*n* = 6 per group). **(K,L)** Representative images of H&E staining of colon sections (*n* = 6 per group). ^ns^*p* > 0.05, *p < 0.05, **p < 0.01, ***p < 0.001, ****p < 0.0001. SAMR1, Senescence-Accelerated Mouse Resistant 1; MR + SAMP8, Senescence-Accelerated Mouse Prone 8 maintained on a 0.17% methionine AIN-93 M diet; MN + SAMP8, Senescence-Accelerated Mouse Prone 8 maintained on a 0.86% methionine AIN-93M diet; MS + SAMP8, Senescence-Accelerated Mouse Prone 8 maintained on a 1.64% methionine AIN-93M diet; H&E, Hematoxylin and Eosin; iFABP, intestinal Fatty Acid-Binding Protein; LPS, Lipopolysaccharide; Zonulin, Zonulin (cleaved form of haptoglobin-2); p16, cyclin-dependent kinase inhibitor 2A; p21, cyclin-dependent kinase inhibitor 1A.

To explore the role of dietary methionine in aging, we conducted an incremental intervention experiment within a safe dose range (Figure 1A). After 4 weeks of intervention, there were no significant differences in the initial and final body weights of mice in each group (Figure 1B, *p* > 0.05), indicating that methionine supplementation had no significant effect on the body weight of SAMP8 mice. Compared with the SAMR1 group, the colon length in the MN + SAMP8 group was significantly shortened (Figures 1C,D). MS + SAMP8 intervention restored colon length to a level significantly higher than N + SAMP8, while MR + SAMP8 had no such effect. ELISA results showed that the levels of intestinal barrier injury markers LPS, IFABP and zonulin in the serum of MN + SAMP8 mice were significantly higher than those in the SAMR1 control group (Figures 1E–G). MS + SAMP8 significantly reduced the above indicators, bringing them close to the SAMR1 level, while MR + SAMP8 did not exert a protective effect. Methionine-supplemented significantly improved intestinal injury in SAMP8 mice. Compared with the SAMR1 group, the aging markers in the MN + SAMP8 group were significantly increased, while those in the MS + SAMP8 group were significantly lower than those in the MN + SAMP8 group. No significant changes were observed in the MR + SAMP8 group (Figures 1H–J). These results indicate that Methionine-supplemented supplementation can alleviate intestinal barrier dysfunction and alleviate aging-related phenotypes. The HE staining results showed that compared with the SAMR1 group, the colonic tissue structure in the MN + SAMP8 group was significantly damaged, manifested as irregular epithelial surface, shortened villi, widened Gruenhagen’s space between the epithelium and lamina lamina, as well as obvious capillary congestion and edema (Figure 1K). Compared with the MN + SAMP8 group, the MR + SAMP8 group still showed dense inflammatory cell infiltration within the lamina lamina under high magnification (Figure 1K). On the contrary, the mucosal epithelium in the MS + SAMP8 group remained intact, the crypt structure was well maintained, no congestion or edema was observed, and the histological injury score was significantly improved (*p* < 0.01). No significant improvement effect was observed in Methionine-restricted supplementation (Figures 1K,L).

### The effects of different doses of methionine on the intestinal mechanical barrier and immune barrier

**Figure 2:**
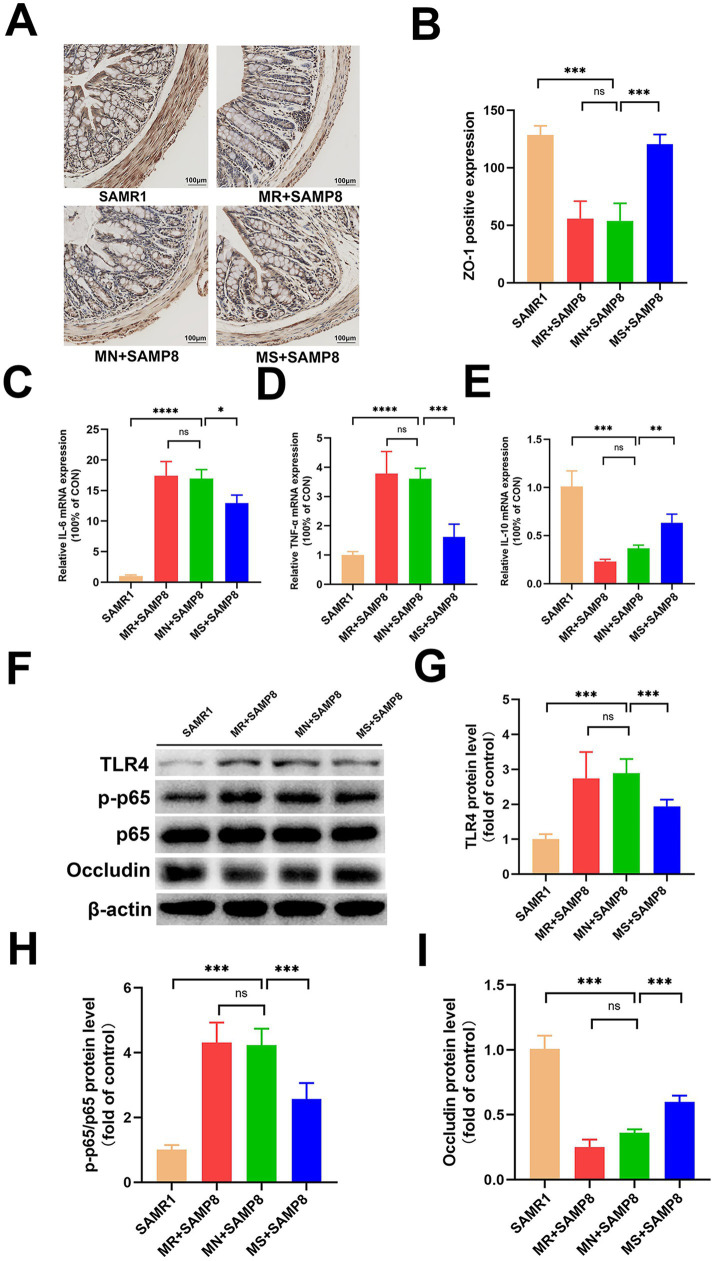
Effects of different methionine doses on the intestinal mechanical barrier. **(A,B)** Relative mean fluorescence intensity of ZO-1 in colonic tissue (*n* = 6 per group). **(C–E)** mRNAlevels of inflammatory cytokines IL-6, TNF-*α* and IL-10 in colon (*n* = 6 per group). **(F–I)** Representative Western blots and quantified protein levels of TLR4, p-p65, and Occludin in colon (*n* = 6 per group). ^ns^*p* > 0.05, *p < 0.05, **p < 0.01, ***p < 0.001, ****p < 0.0001. SAMR1, Senescence-Accelerated Mouse Resistant 1; MR + SAMP8, Senescence-Accelerated Mouse Prone 8 maintained on a 0.17% methionine AIN-93 M diet; MN + SAMP8, Senescence-Accelerated Mouse Prone 8 maintained on a 0.86% methionine AIN-93 M diet; MS + SAMP8, Senescence-Accelerated Mouse Prone 8 maintained on a 1.64% methionine AIN-93 M diet; ZO-1, Zonula Occludens-1 protein; IL*-6, Interleukin-6*; *TNF-α, Tumor Necrosis Factor alpha*; IL*-10, Interleukin-10*; TLR4, Toll-like receptor 4;p-p65, Phospho-NF-κB p65;p65, NF-κB p65; Occludin, a tight-junction integral membrane protein.

Immunohistochemical, Western blot and RT-qPCR analyses showed that compared with the SAMR1 control group, the average fluorescence intensity of ZO-1 in the colon of MN + SAMP8 mice (Figures 2A,B) and the level of Occludin protein were significantly decreased (Figures 2F,I). Meanwhile, the expressions of p-p65 and TLR4 were significantly increased (*p* < 0.001, Figures 2F–H), the mRNA levels of *IL-6* and *TNF-α* were upregulated, while the mRNA level of *IL-10* was downregulated (Figures 2C–E), indicating impaired intestinal barrier integrity accompanied by intensified inflammation. MS + SAMP8 restored the expression of ZO-1 and Occludin, inhibited p-p65 and TLR4, down-regulated the transcription of *IL-6* and *TNF-α,* and up-regulated the mRNA of *IL-10* (Figures 2C–E). On the contrary, MR + SAMP8 did not cause significant changes. In conclusion, Methionine-supplemented supplementation can effectively alleviate age-related intestinal barrier dysfunction and inflammatory responses in SAMP8 mice.

### The effects of different doses of methionine on the fecal microbiota of mice

**Figure 3:**
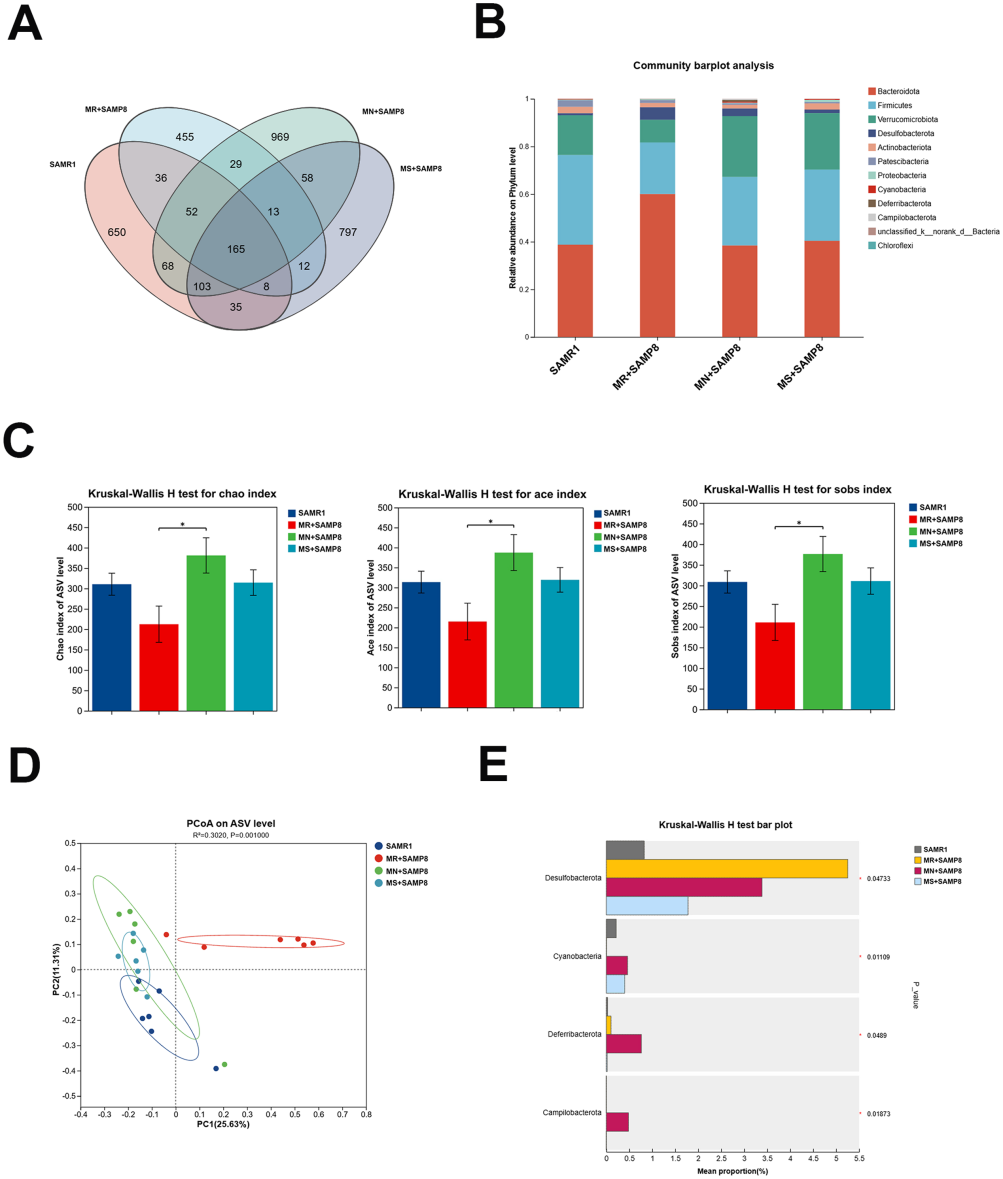
Methionate supplementation restored the disrupted gut microbiota of SAMP8 mice. **(A)** Venn diagram analysis of species. **(B)** phylum–level bacterial community structure across different groups (*n* = 6 per group). **(C)** Gut microbiota diversity using the chao and ace and sobs indices (*n* = 6 per group). **(D)** A clear separation was observed by principal coordinate analysis (PCoA) based on Hellinger (*n* = 6 per group). **(E)** Kruskal–Wallis *H* test bar plot at the Phylum level (*n* = 6 per group). ^ns^*p* > 0.05, *p < 0.05. SAMR1, Senescence-Accelerated Mouse Resistant 1; MR + SAMP8, Senescence-Accelerated Mouse Prone 8 maintained on a 017% methionine AIN-93M diet; MN + SAMP8, Senescence-Accelerated Mouse Prone 8 maintained on a 0.86% methionine AIN-93 M diet; MS + SAMP8, Senescence-Accelerated Mouse Prone 8 maintained on a 1.64% methionine AIN-93 M diet.

In this study, 16S rRNA microbiota diversity analysis technology was adopted to explore the effects of different doses of methionine intervention on the intestinal biological barrier function of SAMP8 mice. The results showed that phylum and genus levels of analysis were conducted on mice in each group. Through 16S rRNA gene sequencing, a total of 1,422,605 high-quality sequences were obtained from 24 samples, with a sequencing depth of 1,000X for each sample (33,404 reads were obtained for each sample). The rarefaction curve reached a plateau, indicating that the sequencing depth covered the vast majority of microorganisms. Cluster analysis obtained 3,450 ASVs within a 97% confidence interval. This study analyzed the species composition of four groups of core ASVs and found that there were a total of 165 common ASVs in the four groups, and the number of ASVs increased after intervention with different doses of methionine (Figure 3A). Among them, the SAMR1, MR + SAMP8, MN + SAMP8, and MS + SAMP8 groups have 650, 455, 969, and 797 unique ASVs, respectively, (Figure 3A). At the door level the top five phyla with the highest abundance are Bacteroidetes, Firmicutes, Verrucomicrobiota, Desulfobacterota and Actinobacteriota. The number of Desulfobacterota decreased after the intervention (Figure 3B). The ACE, Chao, and Sobs *α* -diversity indices of both the MN + SAMP8 group and the MR + SAMP8 group changed significantly (Figure 3C). In the *β* -diversity index analysis, principal coordinate analysis (PCoA) revealed significant differences between the MR + SAMP8 group and the other groups of samples. Figure 3D shows the bacterial community structure of each group at the genus level. Multi-group difference test analysis further identified differentially enriched groups: The Desulfobacterota phylum in the MR + SAMP8 group was significantly elevated. The relative abundances of Cyanobacteria, Campylobacterota and Deferribacterota in the MN + SAMP8 group were significantly higher than those in the other groups (*p* < 0.05, Figure 3E).

**Figure 4:**
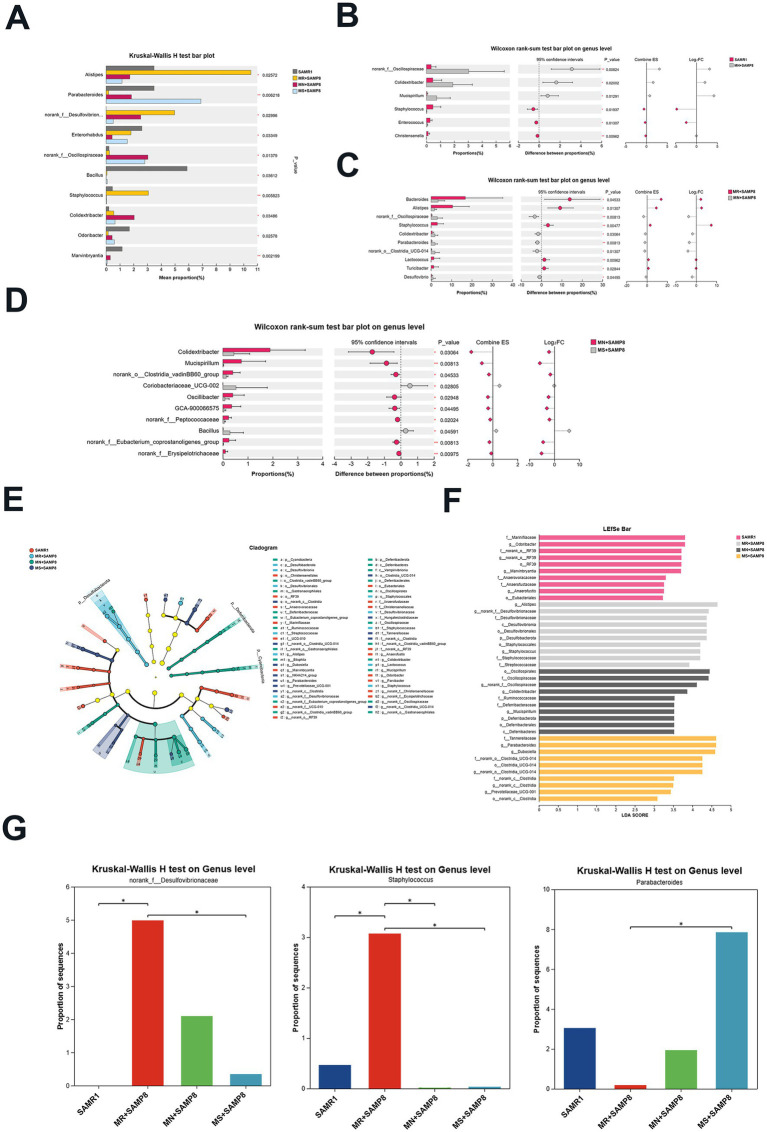
Impact of distinct methionine doses on the murine fecal microbiota. **(A)** Kruskal–Wallis *H* test bar plot at the genus level (*n* = 6 per group). **(B)** The Kruskal–Wallis *H* test bar plot at the genus level of SAMR1 and MN + SAMP8 groups. **(C)** The Kruskal–Wallis *H* test bar plot at the genus level of MR + SAMP8 and MN + SAMP8 groups. **(D)** The Kruskal–Wallis *H* test bar plot at the genus level of MN + SAMP8 and MS + SAMP8 groups. **(E)** LEfSe cladogram (LDA score ≥ 2) based on the Kruskal–Wallis rank-sum test, illustrating differentially abundant bacterial taxa among groups at the genus level. **(F)** Bar graph of linear discriminant analysis (LDA) scores, showing a biomarker taxa (LDA score of >2 and a significance of *p* < 0.05 determined by the Wilcoxon signed–rank test) (*n* = 6 per group). **(G)** Kruskal–Wallis *H* test bar plot at the genus level (*n* = 6 per group). ^ns^*p* > 0.05, *p < 0.05, **p < 0.01, ***p < 0.001, ****p < 0.0001. SAMR1, Senescence-Accelerated Mouse Resistant 1; MR + SAMP8, Senescence-Accelerated Mouse Prone 8 maintained on a 0.17% methionine AIN-93 M diet; MN + SAMP8, Senescence-Accelerated Mouse Prone 8 maintained on a 0.86% methionine AIN-93 M diet; MS + SAMP8, Senescence-Accelerated Mouse Prone 8 maintained on a 1.64% methionine AIN-93 M diet.

Among the TOP10 at the genus level, the abundances of *Alistipes, norank_f__Desulfovibrionaceae* and *Staphylococcus* in the MR + SAMP8 group were significantly higher than those in the other groups. When there is a high-fat diet, a weakened immune system or dysbiosis, *Alistipes* may exacerbate inflammation or metabolic disorders (33, 34). The *Colidextribacter* in the MN + SAMP8 group was significantly increased; *Parabacteroides* in the MS + SAMP8 group increased significantly, while *norank_f__Desulfovibrionaceae* and *Staphylococcus* decreased significantly (Figure 4A). The pin-two comparison results showed that compared with the SAMR1 group, the abundance of *Colidextribacter* in the MN + SAMP8 group was significantly increased, while that in the *Christensenella* group was significantly decreased (Figure 4B). Previous studies have shown that an increase in *Colidextribacter* may lead to an increase in intestinal permeability, thereby triggering an inflammatory response and being associated with intestinal barrier dysfunction (35, 36).

Compared with the MN + SAMP8 group, the relative abundances of *Bacteroides, Alistipes, Oscillospiraceae,* and *Staphylococcus* in the MR + SAMP8 group were significantly increased, while *Parabacteroides* were significantly decreased (Figure 4C). On the contrary, compared with the MN + SAMP8 group, MS + SAMP8 group *Colidextribacter, Clostridia_vadinBB60_group*, *Oscillibacter, GCA-900066575, Peptococcaceae* and The relative abundance of *norank_f__Erysipelotrichaceae* was significantly decreased, while that of *Bacillus* was significantly increased (Figure 4D). It has been reported that *Bacillus* can adjust the balance of gut microbiota by antagonizing pathogenic bacteria and promoting the growth of beneficial bacteria. Thereby alleviating intestinal diseases (37) (Figure 4D).

We used the log₂ fold change (Log₂FC) graph to present all the gut microbiota with an LDA score > 2 (Figure 4E), and listed the top 10 taxonomic units with the highest abundance in each group (Figure 4F). Through linear discriminant analysis (LDA) and effect size (LEfSe) analysis, 10 differentially enriched taxonomic units were identified among the four groups, with an LDA threshold greater than 2. Among them, the SAMR1 group was significantly enriched with *f__Marinifilaceae, Odoribacter, norank_o__RF39, Marvinbryantia,* and *g__Anaerofustis and o__Eubacteriales*. The MR + SAMP8 group was significantly enriched with *Alistipes, Desulfobacterota, norank_f__Desulfovibrionaceae* and *Staphylococcus*. The abundances of *Oscillospirales, Colidextribacter, Ruminococcaceae, Deferribacteraceae* and *Mucispirillum* in the MN + SAMP8 group were higher. The abuntivities of *Parabacteroides, Dubosiella* and *Prevotellaceae_UCG-001* in the MS + SAMP8 group increased significantly. *Parabacteroides* and *Prevotellaceae_UCG-001* not only participate in the metabolic regulation of the host, but also play significant roles in various chronic inflammatory diseases, metabolic syndromes and immune-related diseases (38–40). Furthermore, *Dubosiella* demonstrates potential anti-aging functions by reducing oxidative stress markers (such as malondialdehyde, MDA) and increasing the activity of antioxidant enzymes (such as superoxide dismutase, SOD) (41). The multi-group difference comparison of key bacterial genera showed that there were significant differences between the MR + SAMP8 group and the MS + SAMP8 group in the genera *norank_f__Desulfovibrionaceae, Staphylococcus* and *Parabacteroides*. In the MS + SAMP8 group, the relative abundance of *norank_f__Desulfovibrionaceae* and the potential infectious bacterium *Staphylococcus* significantly decreased, while the relative abundance of the beneficial bacterium *Parabacteroides* significantly increased (Figure 4G).

### Methionine-supplemented diet intervention can improve the overall gut microbiota disorder in SAMP8 mice

**Figure 5:**
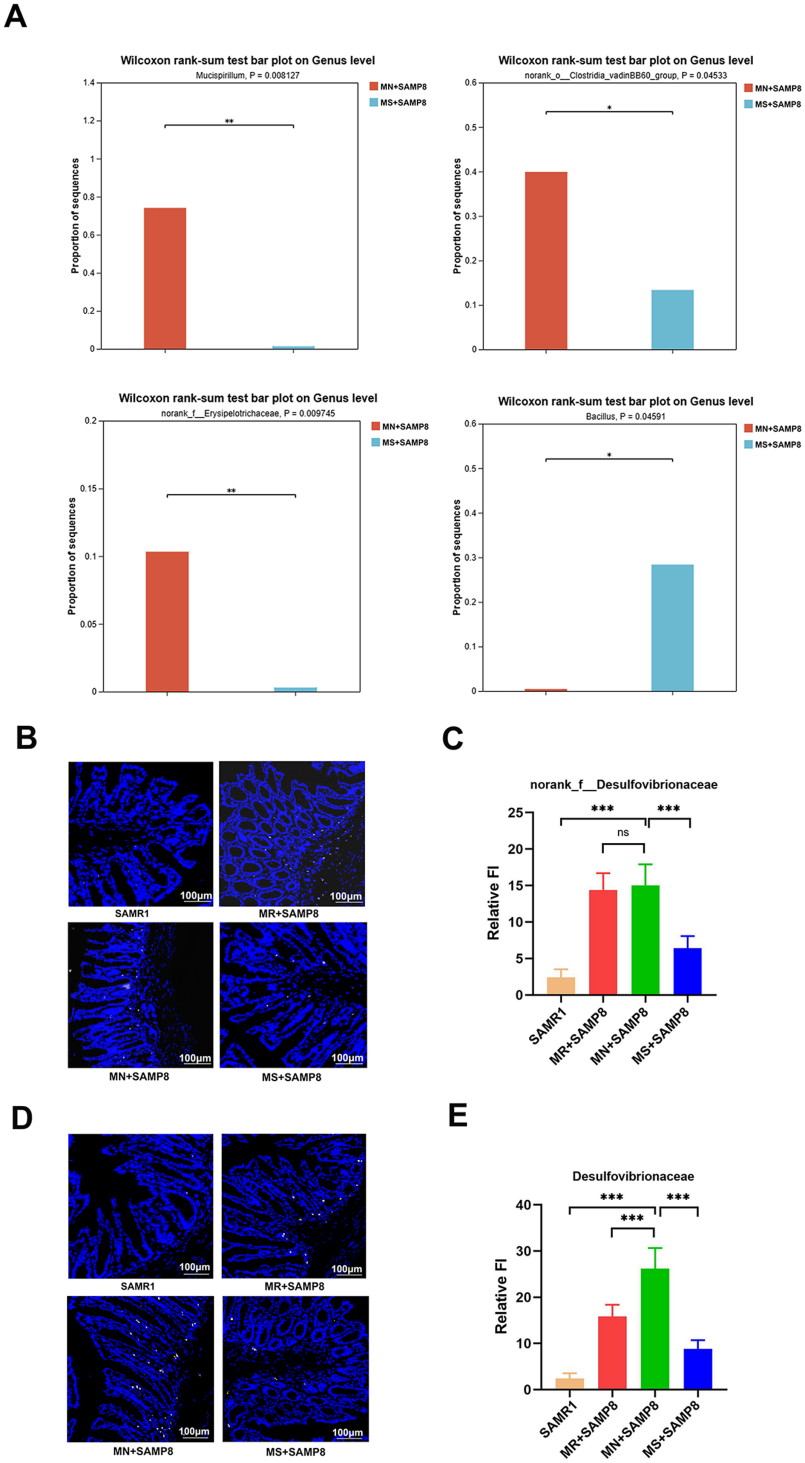
Impact of distinct methionine doses on the fecal microbiota. **(A)** Wilcoxon rank-sum test bar plot at the genus level (*n* = 6 per group). **(B,C)** Probe-based assessment of the spatial distribution of *norank_f__Desulfovibrionaceae* (white). **(D,E)** Probe-based assessment of the spatial distribution of *Desulfovibrionaceae* (yellow). ^ns^*p* > 0.05, *p < 0.05, **p < 0.01, ***p < 0.001, ****p < 0.0001. SAMR1, Senescence-Accelerated Mouse Resistant 1; MR + SAMP8, Senescence-Accelerated Mouse Prone 8 maintained on a 0.17% methionine AIN-93 M diet; MN + SAMP8, Senescence-Accelerated Mouse Prone 8 maintained on a 0.86% methionine AIN-93 M diet; MS + SAMP8, Senescence-Accelerated Mouse Prone 8 maintained on a 1.64% methionine AIN-93M diet.

Compared with the MN + SAMP8 group, the relative abundances of *Mucispirillum, norank_o__Clostridia_vadinBB60_group* and *Erysipelotrichaceae* in the H + SAMP group were significantly decreased, while the relative abundance of *Bacillus* was significantly increased. Studies have shown that *Mucispirillum schaedleri* can affect the integrity of intestinal mucosa and promote inflammation (42). *norank_o__Clostridia_vadinBB60_group* is associated with disruption of intestinal barrier function and enhanced inflammatory response. The abundance of *Erysipelotrichaceae* increases in patients with inflammatory bowel disease (IBD) and has a destructive effect on the intestinal barrier (43) (Figure 5A).

In conclusion, the findings of this study demonstrate that the intestinal microbiota of mice in the MN + SAMP8 group underwent significant changes, the abundance of the *Christensenella* genus was significantly reduced, and the abundance of the *Colidextribacter* genus was significantly increased, leading to increased intestinal permeability and triggering inflammatory responses. After Methionine-restricted intervention, the *g_norank_f__Desulfovibrionaceae* and *Desulfovibrionaceae* in the intestines of SMAP8 mice were significantly enriched. Among them, the genus *Desulfovibrio* generates H₂S through sulfate reduction, which disrupts the intestinal barrier function, enhances the invasiveness of pathogenic bacteria (such as *Escherichia coli*), and indirectly promotes the metabolism of tryptophan/tyrosine (44). Methionine-supplemented intervention promotes the enrichment of beneficial intestinal bacteria, reduces the production of pro-inflammatory cytokines, down-regulates the NF-κB pathway, and exerts a protective effect by regulating T-cell balance and enhancing mucosal barrier function (45).

The spatial distribution of the two bacteria was evaluated, respectively, using*norank_f__Desulfovibrionaceae* (white) and *Desulfovibrionaceae* (yellow) probes. The results showed that the relative abundance of *Desulfovibrionaceae* and *norank_f__Desulfovibrionaceae* in the MN + SAMP8 group increased significantly, while those in the MS + SAMP8 group decreased significantly compared with the MN + SAMP8 group, indicating that Methionine-supplemented improved the abundance of Vibrio desulfuricans (Figures 5B–E).

### Methionine-supplemented improves the intestinal barrier function of SAMP8 mice by regulating MUC2 expression, bacterial ectopic and mucin sulfation

**Figure 6:**
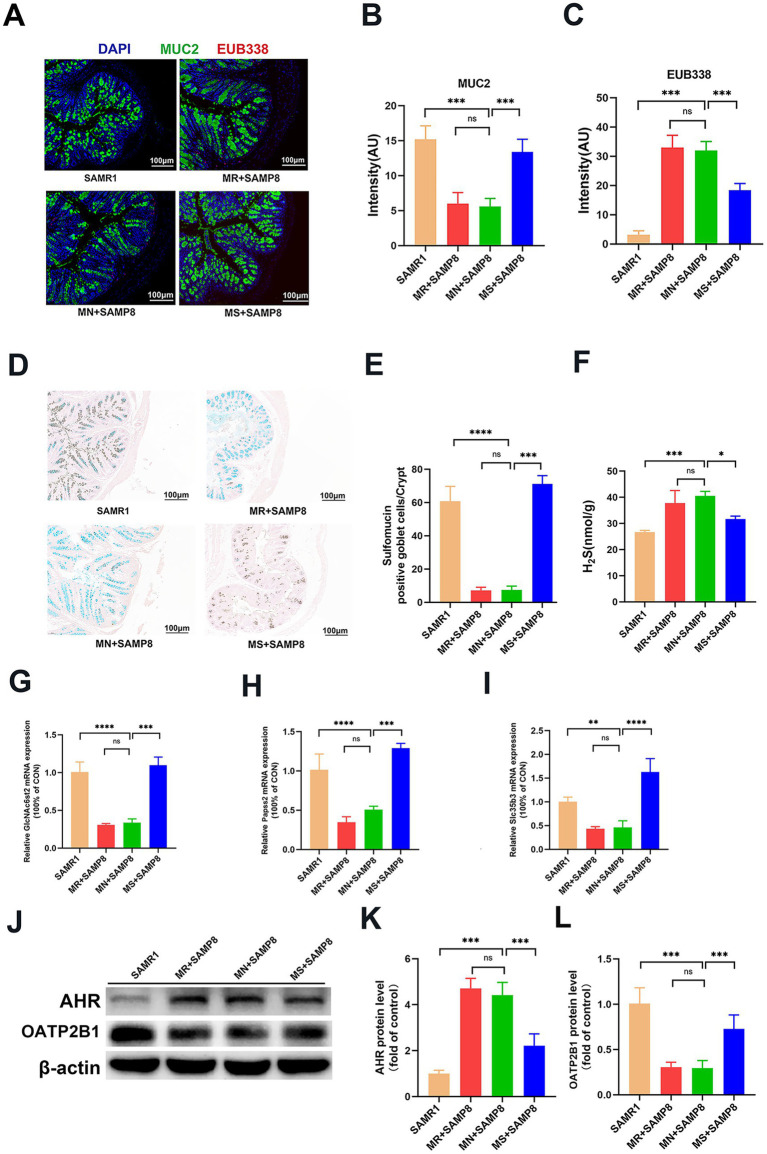
Effects of graded dietary methionine on bacterial translocation, H_2_S concentration, and mucin sulfation in colonic tissue. **(A–C)** Impact of graded methionine intake on MUC2 and EUB338 probe fluorescence intensity in colonic tissue. FISH using EUB338 probe for total bacterial 16S ribosomal RNA (Red) in colon tissue. And the quantification of total number of bacteria/crypt (*n* = 6 per group). **(D,E)** Representative micrographs of HID staining and sulfomucin layer quantification (*n* = 6 per group). **(F)** The contents of H_2_S in colon tissue (*n* = 6 per group). **(G–I)** The relative colonic mRNA expression of *GlcNAc6st2, Papss2,* and *Slc35b3*. **(J-L)** Western Blotting of AHR and OATP2B1 in the colon (*n* = 6 per group). ^ns^*p* > 0.05, *p < 0.05, **p < 0.01, ***p < 0.001, ****p < 0.0001. SAMR1, Senescence-Accelerated Mouse Resistant 1; MR + SAMP8, Senescence-Accelerated Mouse Prone 8 maintained on a 0.17% methionine AIN-93 M diet; MN + SAMP8, Senescence-Accelerated Mouse Prone 8 maintained on a 0.86% methionine AIN-93 M diet; MS + SAMP8, Senescence-Accelerated Mouse Prone 8 maintained on a 1.64% methionine AIN-93 M diet; H_2_S, Hydrogen sulfide; G*lcNAc6st2*, *N-acetylglucosamine-6-O-sulfotransferase 2*; *Papss2*,*3′-phosphoadenosine-5′-phosphosulfate synthase 2; Slc35b3, solute carrier family 35 member b3; AHR*, Aryl Hydrocarbon Receptor; OATP2B1, Solute Carrier Organic Anion Transporter Family Member 2B1.

MUC2 is the main structural component of the intestinal mucus layer. It directly regulates intestinal permeability by maintaining the integrity of the mucus layer, regulating the glycosylation process, and having a synergistic effect with tight junctions (TJs) (46). We labeled MUC2 with green fluorescent probes and detected the integrity of the mucus layer in the intestinal epithelial layer of mice through fluorescence *in situ* hybridization (FISH) technology. The results showed that compared with the SAMR1 group, the green fluorescence signal of MUC2 in MN + SAMP8 mice was significantly weakened. Compared with the MN + SAMP8 group, the green fluorescence signal in the colon of the MS + SAMP8 group was significantly enhanced, indicating that the colonic mucus expression of mice in the SAMP8 group was significantly improved (Figures 6A,B). To analyze the impact of aging on epithelial permeability, we used a broad-spectrum bacterial probe (EUB338) to detect bacteria in the intestinal epithelial layer of mice through fluorescence in situ hybridization (FISH). The results showed that intestinal bacterial translocation occurred in MN + SAMP8 and MR + SAMP8 mice, and obvious red fluorescent probe signals could be seen in the intestines. Compared with the SAMR1 group, the total number of bacteria and their migration to the deep mucosa in MN + SAMP8 mice were significantly increased. Compared with the MN + SAMP8 group, the fluorescence signal in the colon of the MS + SAMP8 group was reduced, and the total number of ectopic bacteria in the SAMP8 group mice was significantly decreased (Figures 6A,C). These results indicate that aging leads to the destruction of the intestinal epithelial barrier, while Methionine-supplemented intervention can improve bacterial ectopic conditions.

The HID-AB staining results showed that the sulfation level of intestinal epithelium in the MN + SAMP8 group was significantly reduced, while the area of colonic thiosin increased significantly after Methionine-supplemented intervention (Figures 6D,E). The H_2_S content in the intestinal tissue of mice in the MN + SAMP8 group was significantly higher than that in the SAMR1 group. Compared with the MN + SAMP8 group, the H_2_S content in the MS + SAMP8 group was significantly decreased, while there was no significant difference in the MR + SAMP8 group (Figure 6F). RT-qPCR analysis indicated that the mRNA levels of mucus sulfation-related genes *GlcNAc6st2, Papss2* and *Slc35b3* in the intestinal tissues of mice in the MS + SAMP8 group were significantly increased (Figures 6G–I). These results indicate that Methionine-supplemented can promote the sulfation level of mucin in the intestinal tissue of SAMP8 mice and improve colonic injury. In addition, MS + SAMP8 treatment significantly increased the expression of OATP2B1 protein and the content of AHR protein in the colon of mice (Figures 6J–L). OATP2B1 is a major drug transporter that transports various small molecules in the intestinal tract. The Methionine-supplemented group promotes the transport of toxic substances (such as phenol sulfate, Indoxyl sulfate, phenylglucuronide, etc.) by increasing the expression of transport proteins, and simultaneously raises the mRNA expression levels of intestinal sulfation indicators *GlcNAc6st2, Papss2* and *Slc35b3*, thereby promoting intestinal mucin sulfation. Significantly improve intestinal damage in SAMP8 aging mice.

## Discussion

Aging is a complex process of multi-system functional decline, among which the deterioration of intestinal health is regarded as the core factor driving systemic low-grade inflammation and various age-related diseases (13). At present, intervention strategies for delaying aging include drug therapy, lifestyle adjustment, nutritional supplementation, gene therapy and cell therapy (47–50). Among these, plant compounds with broad-spectrum antioxidant and anti-inflammatory properties, as well as strategies for improving metabolic health by regulating macronutrient intake, have attracted much attention due to their high safety (51, 52). Specific amino acid supplements (such as leucine) have been proven to effectively alleviate sarcopenia in the elderly, highlighting the potential of targeted nutritional intervention in addressing specific aging phenotypes (53). However, the specific effects of essential amino acids (such as methionine) on aging-related intestinal barrier dysfunction and their dose effects remain poorly studied.

Our research indicates that a methionine-supplemented diet (1.64%) can significantly reverse the expression of aging factors and intestinal barrier dysfunction in accelerated aging mice (SAMP8), while a methionine-restricted diet (0.17%) conferred no observable benefit. This protective effect is manifested on multiple levels. Firstly, a methionine-supplemented diet significantly down-regulates the expression of aging markers p16 and p21 in colon tissue. This finding is highly consistent with the goals of many anti-aging interventions, as eliminating p16/ p21-positive senescent cells is an effective strategy to delay the decline of tissue function (54). In addition, a methionine-supplemented diet reduced the levels of LPS, IFABP and Zonulin in the serum, which are key biomarkers of increased intestinal permeability and systemic inflammation (27). Histological and immunofluorescence analyses further confirmed that methionine-supplemented restored the structural integrity of the colon and upregulated the expression of tight junction proteins ZO-1 and Occludin. Most importantly, we have revealed an unreported mechanism that methionine-supplemented increases the host’s intestinal mucus sulfation capacity by regulating the intestinal microbiota, especially by inhibiting H₂S-producing bacteria, which constitutes the “microbiota-sulfation axis” we proposed. This study systematically reveals a potential new mechanism through which methionine-supplemented dietary methionine effectively improves the intestinal barrier of aging mice by reshaping the structure of the intestinal microbiota and promoting sulfation of the host’s intestinal mucus, providing a brand-new molecular framework for understanding the metabolic crosstalk between diet, microorganisms and host aging.

The results of this study are consistent with existing literature, confirming that aging is closely related to weakened intestinal barrier function (i.e., “leaky gut”). Weakened intestinal barrier function leads to the translocation of microbial products (such as LPS), activating the TLR4/NF-κB inflammatory pathway and becoming a key driver of “inflammatory aging” (55). Our research found that this pathway was activated in SAMP8 mice, and methionine-supplemented diet could effectively inhibit this process, with an effect similar to that of anti-inflammatory substances such as plant compounds (56). The innovation of this study lies in the fact that for the first time, the protective effect of methionine is directly associated with its specific dose and the regulation of microbial metabolism. Previous studies have mostly focused on the benefits of methionine restriction (MR) for extended lifespan (57, 58), but the impact of MR On the intestinal barrier is controversial. The results of this study indicate that Methionine-supplemented supplementation within a safe range may be a more direct and targeted strategy for protecting the intestinal barrier in the elderly. This study elucidates a novel mechanism by which methionine affects host health by regulating sulfur metabolism mediated by the gut microbiota. Research has found that a methionine-supplemented diet significantly alters the structure of the gut microbiota, specifically manifested as an increase in the abundance of potential beneficial bacteria genera (such as *Parabacteroides*), as well as sulfate-reducing bacteria (SRB) that produce H₂S. In particular, the abundance of the genus *norank_f__Desulfovibrionaceae* decreased significantly. Studies have shown that *Desulfovibrionaceae* can disrupt the intestinal epithelial barrier and exacerbate inflammatory bowel disease (IBD) by reducing dietary sulfates to cytotoxic H₂S (59, 60). Under normal circumstances, the colon absorbs approximately 340 micromoles of sulfides every day. If the detoxification function of the colonic mucosa is impaired, this concentration can cause local and systemic damage (61).

Our research indicates that a methionine-supplemented diet significantly inhibits the proliferation of *Desulfovibrionaceae* and *norank_f__Desulfovibrionaceae* and reduces the level of H₂S in colon tissue. This indicates that methionine-supplemented may inhibit the ecological niche of SRB by altering the intestinal microenvironment or providing competitive substrates. This inhibitory effect on the metabolism of harmful microorganisms provides a new perspective for the protection of the intestinal barrier. Furthermore, for the first time, we directly correlated the regulation of sulfur metabolism by the microbiota with the sulfation capacity of the host’s intestinal tract. The sulfation process of intestinal epithelial cells, especially that of mucin, is crucial for the formation of a negatively charged mucus layer and the physical rejection of bacteria (62). We found that a methionine-supplemented diet not only inhibited the microbiota that converts sulfate to H₂S, but also significantly upregulated the expression of key genes in the host sulfation pathway (such as *Papss2*), and increased the production of sulfomucin in the colon. This indicates that by inhibiting the consumption of sulfate by microorganisms, a methionine-supplemented diet “releases” sulfate, enabling it to be utilized more effectively by the host and thereby strengthening the chemical barrier. Meanwhile, the expression of aromatic hydrocarbon receptor (AHR) and transport protein (OATP2B1) was upregulated, suggesting that methionine-supplemented may synergistically protect intestinal homeostasis by enhancing the intestinal perception and detoxification ability of microbial metabolites. The discovery of this “microbiota-sulfation axis” may provide basic data for clinical trials of methionine supplementation in elderly people at risk of intestinal permeability problems. Although this study has made important discoveries, there are still some limitations and it points out the direction for future research. Firstly, this study employed the SAMP8 accelerated aging model, and the universality of its results in both natural aging models and humans remains to be further verified. Secondly, although we have revealed a strong correlation between the changes in the microbiota and the recovery of sulfation, most intestinal microbes are difficult to isolate and culture. Therefore, we cannot ultimately confirm the causal relationship through experiments using germ-free mice to colonize specific strains (such as *norank_f__Desulfovibrionaceae*), and further exploration is needed.

In conclusion, this study demonstrates that a methionine-supplemented diet alleviates intestinal barrier dysfunction and aging during the aging process by regulating the microbiota, sulfur metabolism, inflammation, and mucous sulfation pathways. These findings not only deepen our understanding of the role of amino acids in intestinal health and aging, but also propose a promising and targeted nutritional intervention strategy, with the aim of promoting healthy aging by improving intestinal health.

## Data Availability

The datasets presented in this study are publicly available. This data can be found at: https://www.ncbi.nlm.nih.gov/, accession number PRJNA1314752.
